# Supplementary material for: Switchable stimulated Raman scattering microscopy with photochromic vibrational probes
Source: Nat Commun. 2021 May 25;12:3089. doi: 10.1038/s41467-021-23407-2 (PMC8149663; doi:10.1038/s41467-021-23407-2)
Supplement: Supplementary file 1 — Supplementary Information [file 41467_2021_23407_MOESM1_ESM.pdf]

Switchable stimulated Raman scattering microscopy with photochromic vibrational probes

Jianpeng Ao[1‡], Xiaofeng Fang[2‡], Xianchong Miao[1], Jiwei Ling[1], Hyunchul Kang[3], Sungnam Park[3], Changfeng Wu[2*], and Minbiao Ji[1*]

[1]*State Key Laboratory of Surface Physics and Department of Physics, Human Phenome Institute, Multiscale Research Institute of Complex Systems, Academy for Engineering and Technology, Key Laboratory of Micro and Nano Photonic Structures (Ministry of Education), Fudan University, Shanghai 200433, China*

[2]*Department of Biomedical Engineering, Southern University of Science and Technology, Shenzhen 518055, China*

[3]*Department of Chemistry and Research Institute for Natural Science, Korea University, Seoul, 02841, Korea*

‡ *These authors contributed equally.*

*Corresponding authors: minbiaoj@fudan.edu.cn, wucf@sustech.edu.cn

Supplementary Information

Supplementary note 1 – synthesis of DTE-Alkyne derivatives.

Materials

Tetrahydrofuran (THF), dichloromethane (DCM) methanol, acetone, acetonitrile, acetic acid, triethylamine (Et3N), sodium hydroxide, and diethel ether were purchased from Beijing Chemical Plant. Sodium hexafluorophosphate, iodobenzene, Pd(PPh3)4, trimethylsilylacetylene, n-BuLi (2.5 M, Hexane solution), perfluorocyclopentene, copper(I) iodide, iodoethane, and 4-(4-iodophenyl)pyridine were purchased from J&K Chemical Ltd. (Beijing, China). Poly (styrene-comaleic anhydride) (PSMA, cumene terminated, average MW ≈ 1700, styrene content 68%), N-bromosuccinimide, CD3OD, and CDCl3 were purchased from Sigma-aldrich. All the above chemicals were of analytical grade and used as received without further purification. THF was distilled in the presence of sodium benzophenone under protection of dry nitrogen prior to use.

Instruments

^{1}H NMR spectra were recorded on a Bruker Avance 400 MHz spectrometer using CDCl3 or DMSO-d6 as solvent and tetramethylsilane as an internal standard (d= 0.00 ppm). UV-Vis spectra were measured on a Shimadzu UV-2550 spectrophotometer.

Synthesis

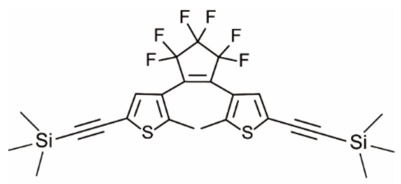

DTE-TMS

The synthesis of all compounds is according to literatures 1-3. ^{1}H NMR (400 MHz, CDCl3) δ 7.20 (s, 2H), 1.87 (s, 6H), 0.25 (s, 18H). MS: m/z calcd 560.01; found, 559.01.

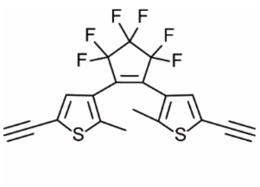

DTE-alkyne

DTE-TMS (1 eq, 0.79 g) was dissolved into methanol/THF (4:1, v:v) mixture under stirring. Then, NaOH (10 eq, 0.6 g) was added and the mixture was stirred at room temperature for 12 h. The resulting mixture was extracted with DCM for three times. The combined organic layer was washed by water, and dried with anhydrous Na_2SO_4. The solvent was evaporated under reduced pressure. The crude product was simply purified by column chromatography over silica gel with hexane as the eluent to yield a white solid (0.39 g, yield: 66.7%). ^1H NMR (400 MHz, $CDCl_3$) δ 7.23 (s, 2H), 3.36 (s, 2H), 1.90 (s, 6H). MS: m/z calcd 416.01; found, 415.01.

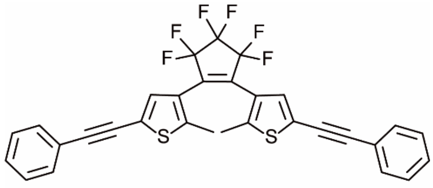

DTE-Ph

DTE-alkyne (1 eq, 89.6 mg) and iodobenzene (3 eq, 0.1 mL) were dissolved in 50 mL Et_3N and THF mixture (1:1, v:v). The mixture was degassed by Ar for 30 min, $Pd(PPh_3)_4$ (5 mg) and copper(I) iodide (2 mg) were added. The reaction mixture was stirred at 70°C for 12 h under Ar atmosphere. After cooling to room temperature, the solvent was evaporated under reduced pressure. The residue was extracted with DCM. The organic layer was washed with HCl (1 M) and water, and then dried over anhydrous Na_2SO_4. The solvent was evaporated under reduced pressure. The crude product was simply purified by column chromatography over silica gel with hexane as the eluent to

yield a white solid (92.5 mg, yield: 75.7%). ^{1}H NMR (400 MHz, $CDCl_3$) δ 7.51 (dd, J = 6.4, 3.0 Hz, 4H), 7.37 – 7.33 (m, 6H), 7.26 – 7.24 (m, 2H), 1.95 (s, 6H). MS: m/z calcd 568.08; found, 567.07.

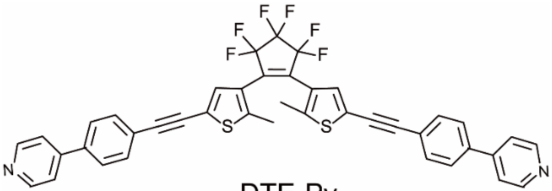

DTE-Py

The synthesis of DTE-Py is the same to the DTE-Ph. DTE-alkyne (1 eq, 80 mg) and 4-(4-iodophenyl) pyridine (3 eq, 133 mg) were used. The crude product was simply purified by column chromatography over silica gel with methanol/DCM mixture (1:120, v:v) as the eluent to yield a black green solid (62 mg, yield: 44.5%). ^{1}H NMR (400 MHz, $CDCl_3$) δ 8.12 – 7.38 (m, 16H), 7.32 (s, 2H), 1.98 (s, 6H). MS: m/z calcd 722.12; found, 723.14.

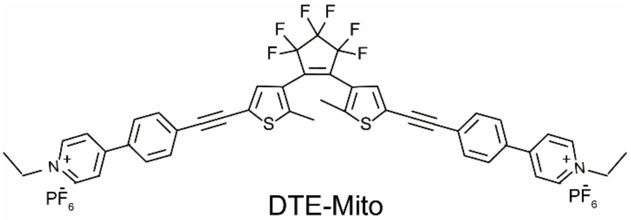

DTE-Mito

DTE-Py (1 eq, 38.4 mg) was dissolved into a 50 mL two-necked round bottom flask with 10 mL acetonitrile. Iodoethane (5 eq, 0.02 mL) was added and the mixture was heated to 80°C for overnight. After cooling to room temperature, 20 mL diethyl ether was added. The black green solids were filtered and washed with diethyl ether. The solids were dissolved in 5 mL methanol/acetone mixture (1:5, v:v) and mixed with saturated $NaPF_6$ solution (5 mL). After stirring for 1 h, solvent was evaporated. The solids were filtered off again, washed by water and dried under vacuum at 60°C for 24

h to yield a black green solid (43.7 mg, yield: 76.9%). ^{1}H NMR (400 MHz, CD_3OD) δ 8.97 (d, J = 6.9 Hz, 4H), 8.42 (d, J = 6.9 Hz, 4H), 8.05 (d, J = 8.5 Hz, 4H), 7.77 (d, J = 8.5 Hz, 4H), 7.40 (s, 2H), 4.67 (d, J = 7.4 Hz, 4H), 2.02 (s, 6H), 1.68 (t, J = 7.4 Hz, 6H). MS: m/z calcd 780.21; found, 390.10.

Supplementary note 2 – Photo-cyclization conversion ratio measurement.

The area of spontaneous Raman peak is proportional to the content of substance[4]. Therefore, the conversion efficiency of the DTE-Ph is estimated from the Raman spectra intensity variation.

$$\emptyset_{con} = \frac{\int \Delta I \mathrm{d}k}{\int I \mathrm{d}k}$$

Where $\emptyset_{con}$ is the conversion efficiency, ΔI is the intensity variation from open-form to closed-form and I is the original intensity of open-form at a certain wavenumber, dk is the wavenumber interval.

The conversion efficiency estimation in SRS is more complication due to the lower spectral resolution, so that we need to decompose the SRS spectra. First, the Voigt fitting of the spectrum of layer1 without UV irradiation (Fig. 2a-b, Supplementary Fig. 8) gave us the peak position and peak width parameter of open-form. Second, through two Voigt fitting of layer4 while fixing the parameter of open-form, we can derive the peak position and peak width parameter of closed-form. Third, fitting the spectra of all layers with derived parameter, we can calculate each composition percentage (Supplementary Table 1). We can also derive the ratio of SRS intensity of closed-form to open-form and the results from all layers match well.

Supplementary Figures

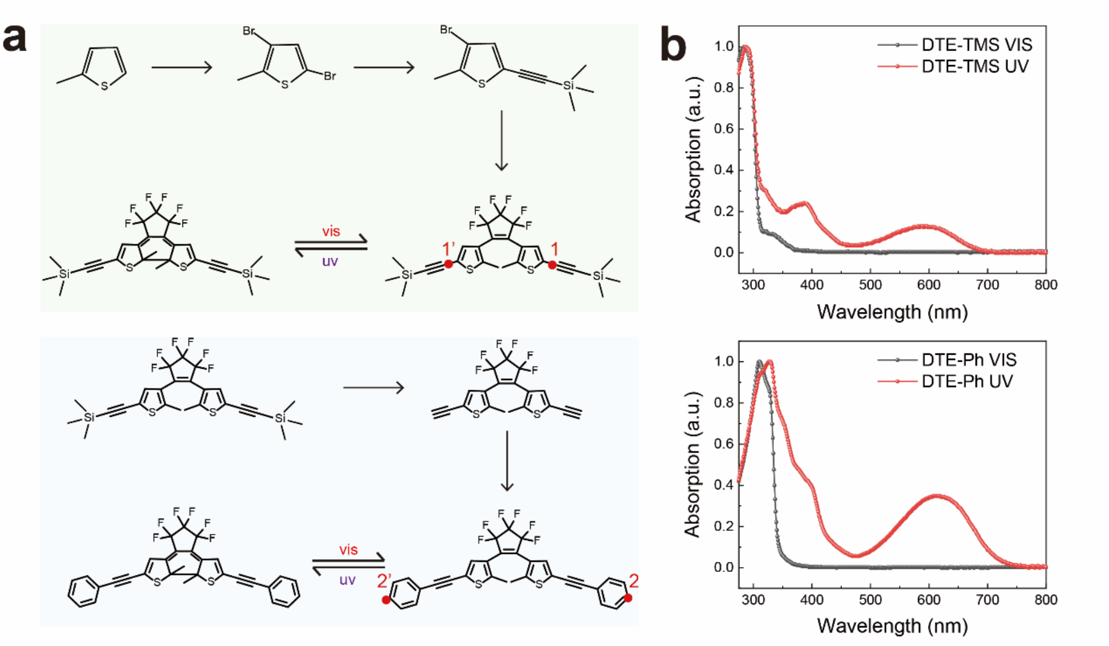

Supplementary Fig. 1. Photo-switching properties of DTE-TMS and DTE-Ph. (a) The synthesis route of DTE-TMS and DTE-Ph; (b) Absorption spectra of DTE-TMS and DTE-Ph after visible or UV irradiation.

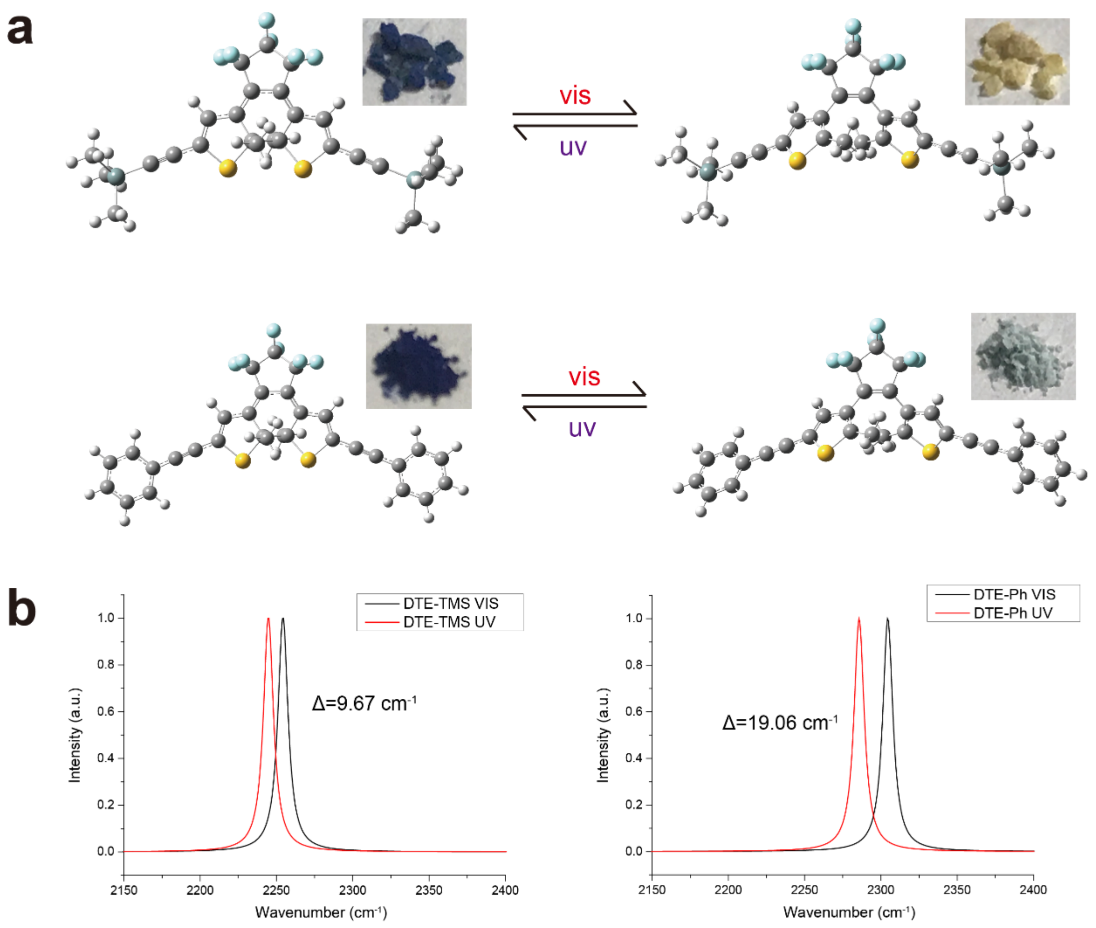

Supplementary Fig. 2. DFT calculation results of DTE-TMS and DTE-Ph. (a) 3D structure and (b) Raman frequency of DTE-TMS and DTE-Ph calculated by Gaussian under B3LYP functional / 6-31g(d) basis set.

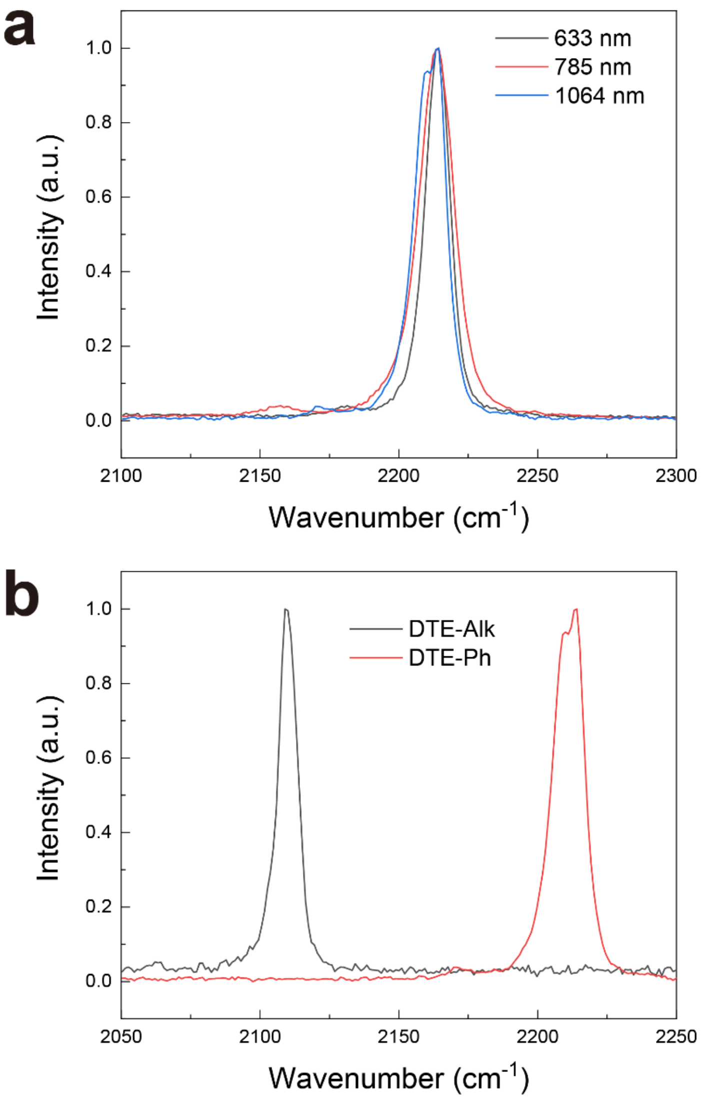

Supplementary Fig. 3. Spontaneous Raman spectra of DTE-Ph. (a) Spontaneous Raman spectra of DTE-Ph measured with 633nm (FWHM ~ 9 cm^{-1}), 785nm (FWHM ~13 cm^{-1}) and 1040nm (FWHM ~ 7 cm^{-1} for each peak) excitation. (b) Verification of the small "splitting" appeared in 1064 nm pumped Raman spectroscopy. The DTE-Alk molecule doesn't show any spectral splitting (FWHM ~ 7 cm^{-1}), excluding systematic error of the Raman spectrometer.

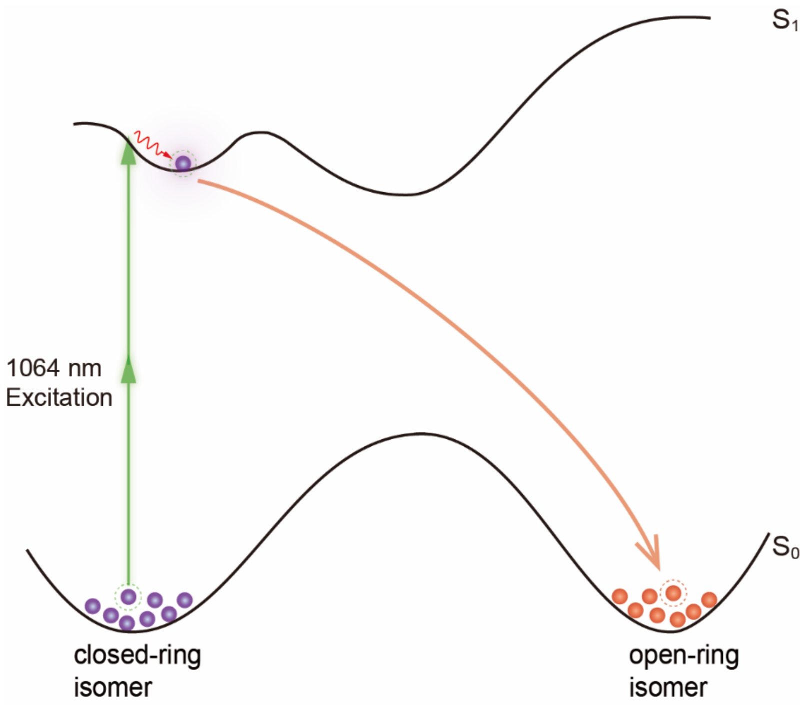

Supplementary Fig. 4. Two-photon excited photo-cycloreversion of continuous 1064 nm irradiation during spontaneous Raman measurements, resulting in partial photo-cyclization after UV irradiation.

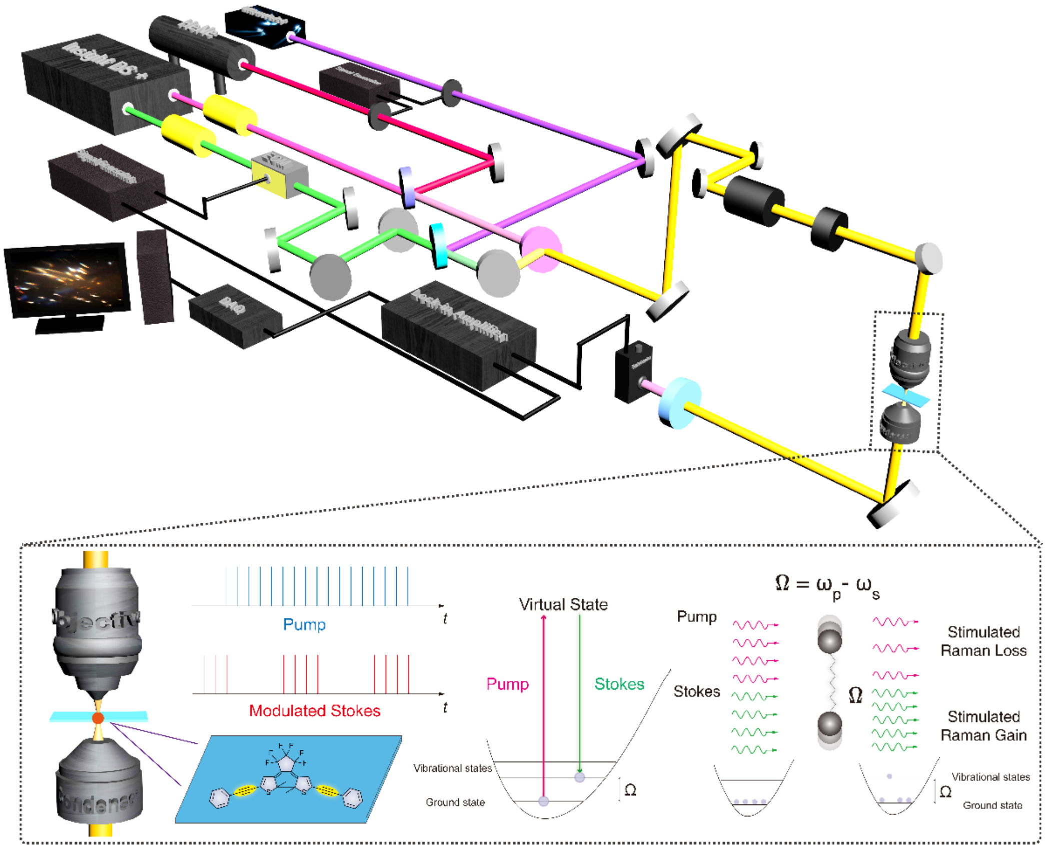

Supplementary Fig. 5. Optical layout of the experimental apparatus.

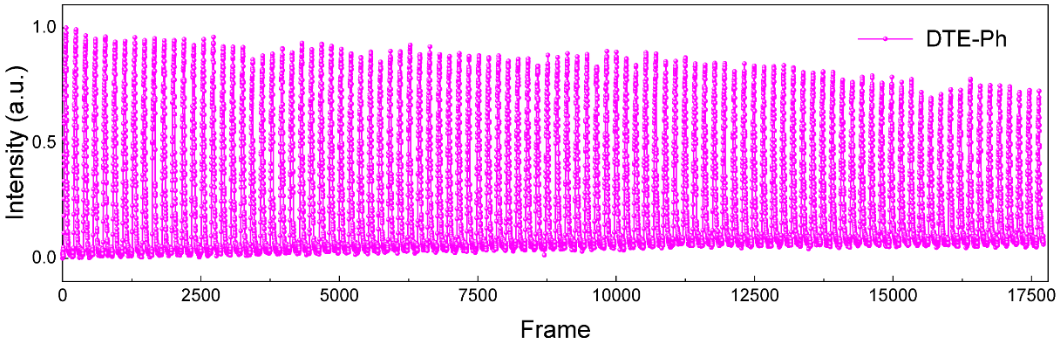

Supplementary Fig. 6. The fatigue feature of DTE-Ph, representing 100 full conversion cycles (over 17500 frames, 28 ms/frame, 16 × 12 pixels).

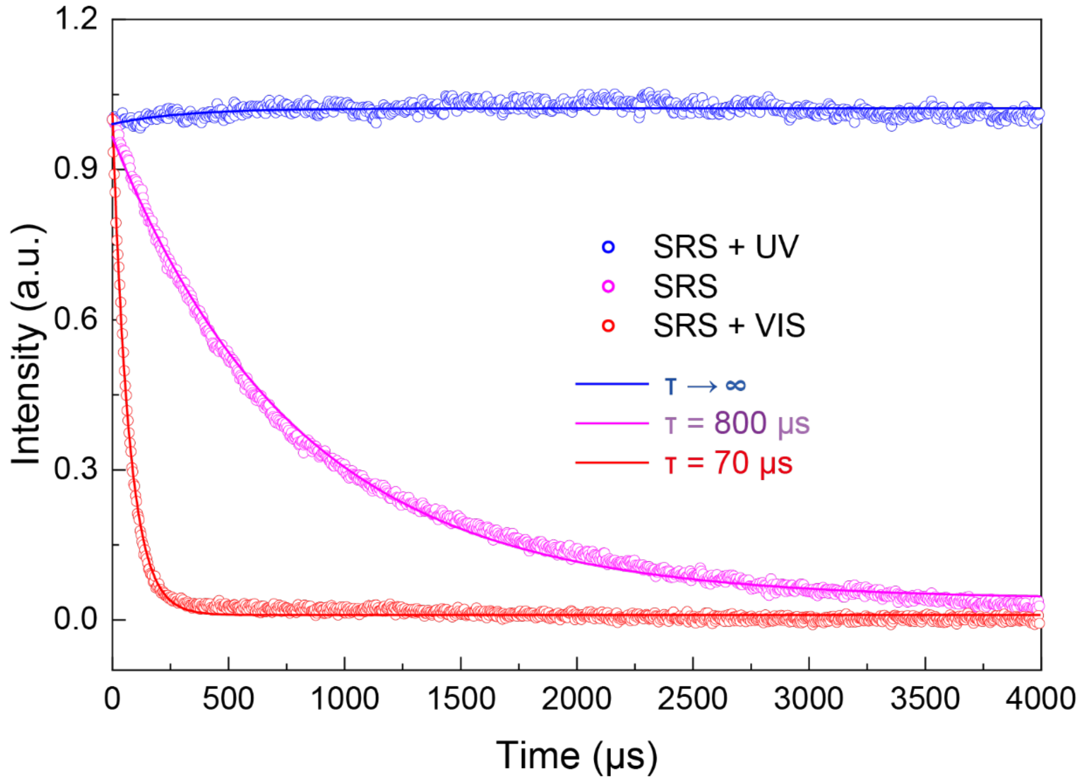

Supplementary Fig. 7. Stability characterization of the photoactive Raman peak (2194 cm^{-1}) under different excitation conditions during SRS imaging. With normal SRS condition, only the pump and Stokes beams interact with the sample, the SRS signal shows a slow decay after ~ 400 image frames (2 μs/pixel). With an additional visible beam, the SRS signal is quenched ~ 10 times faster. Whereas with UV beam, the SRS signal stays almost persistent.

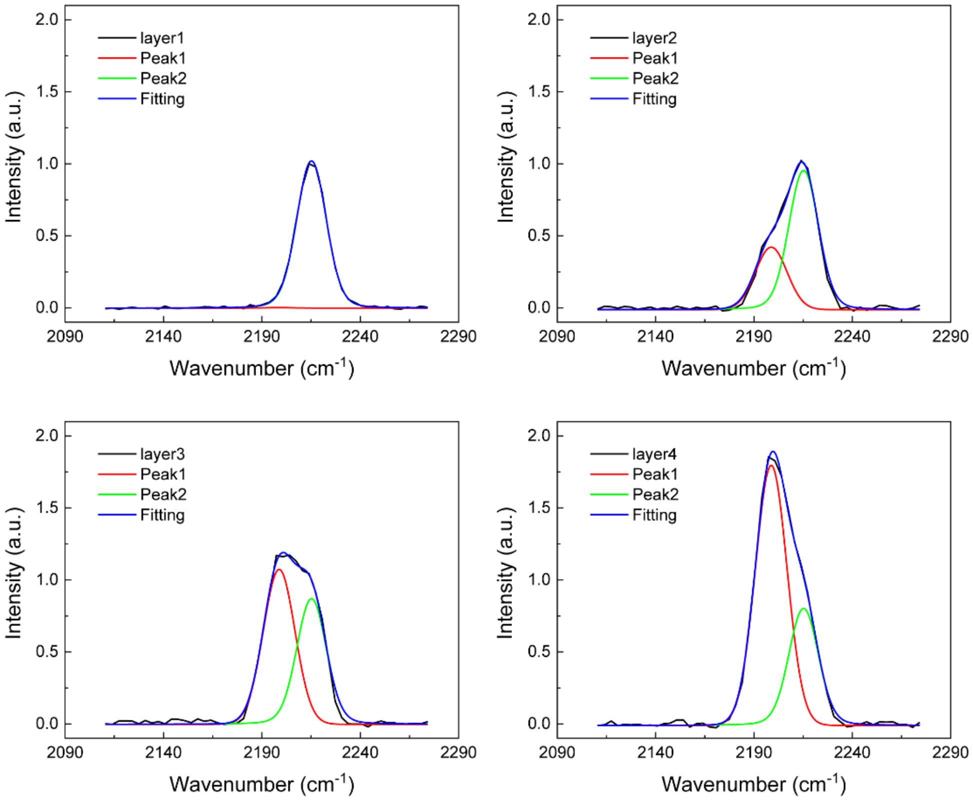

Supplementary Fig. 8. Spectral decomposition analysis of the SRS spectra of DTE-Ph PMMA film under different UV irradiation time (Layer 1: 0 μs; Layer 2: 50 μs; Layer 3: 100 μs; Layer 4: 150 μs).

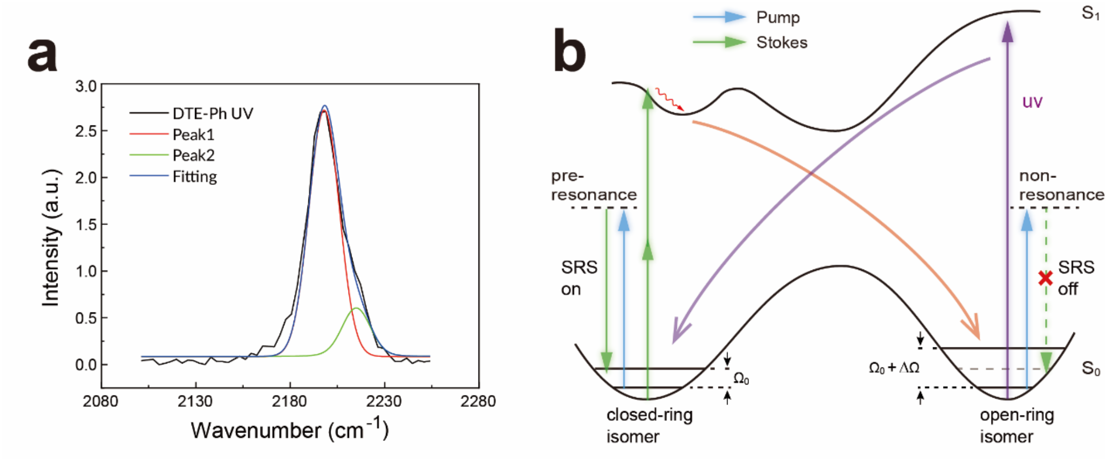

Supplementary Fig. 9. The equilibrium between continuous UV and pump, Stokes irradiation. (a) Spectral decomposition analysis of the SRS spectra of DTE-Ph in Fig. 1c. (b) Schematic diagram of the equilibrium between UV photo-cyclization and SRS two-photon induced photo-cycloreversion.

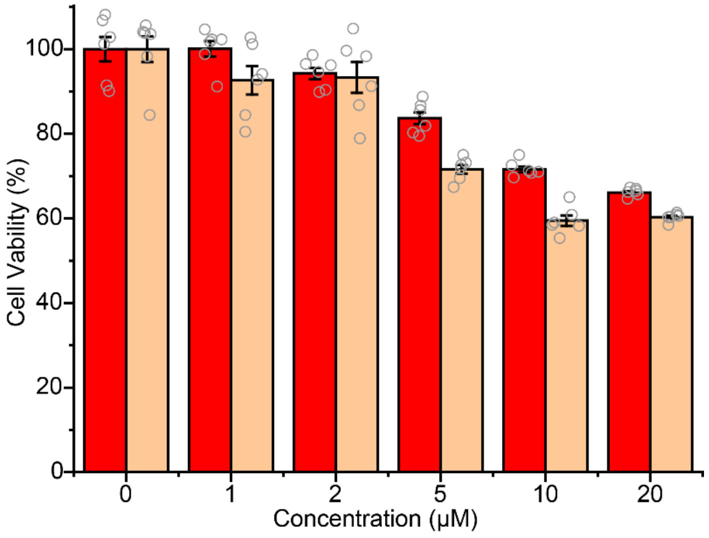

Supplementary Fig. 10. Cell viability tested by MTT assay of DTE-Ph-Mito cultured HeLa cells (n=6 biologically independent samples, data shown as mean ± SD).

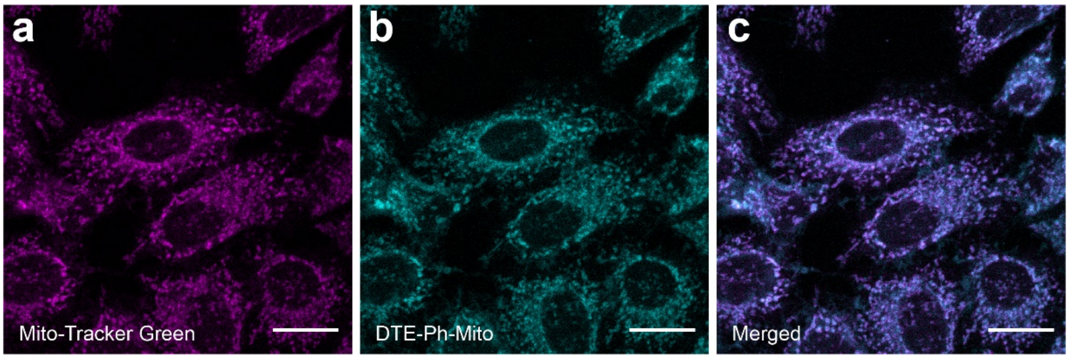

Supplementary Fig. 11. Mitochondrial labeling specificity of DTE-Ph-Mito. Two-photon fluorescent images of HeLa cells co-stained with (a) Mito-Tracker Green (50 nM) and (b) DTE-Ph-Mito (4 μM) in culture medium for 20 min. (c) Merged image of (a) and (b). Excitation wavelength 976 nm for Mito-Tracker Green and 801 nm for DTE-Ph-Mito. Scale bar: 20 μm.

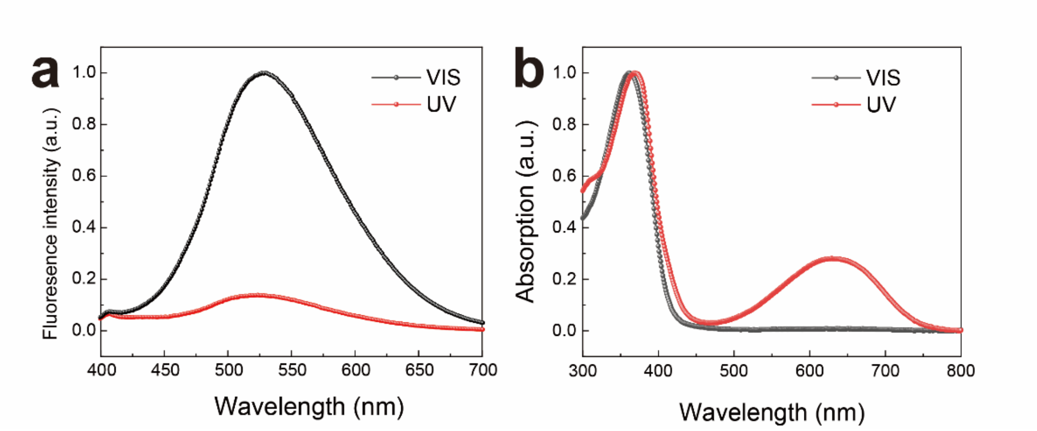

Supplementary Fig. 12. Photophysical properties of DTE-Ph-Mito. (a) The fluorescence (excitation wavelength: 362 nm) and (b) absorption spectra of DTE-Ph-Mito after UV and visible irradiations.

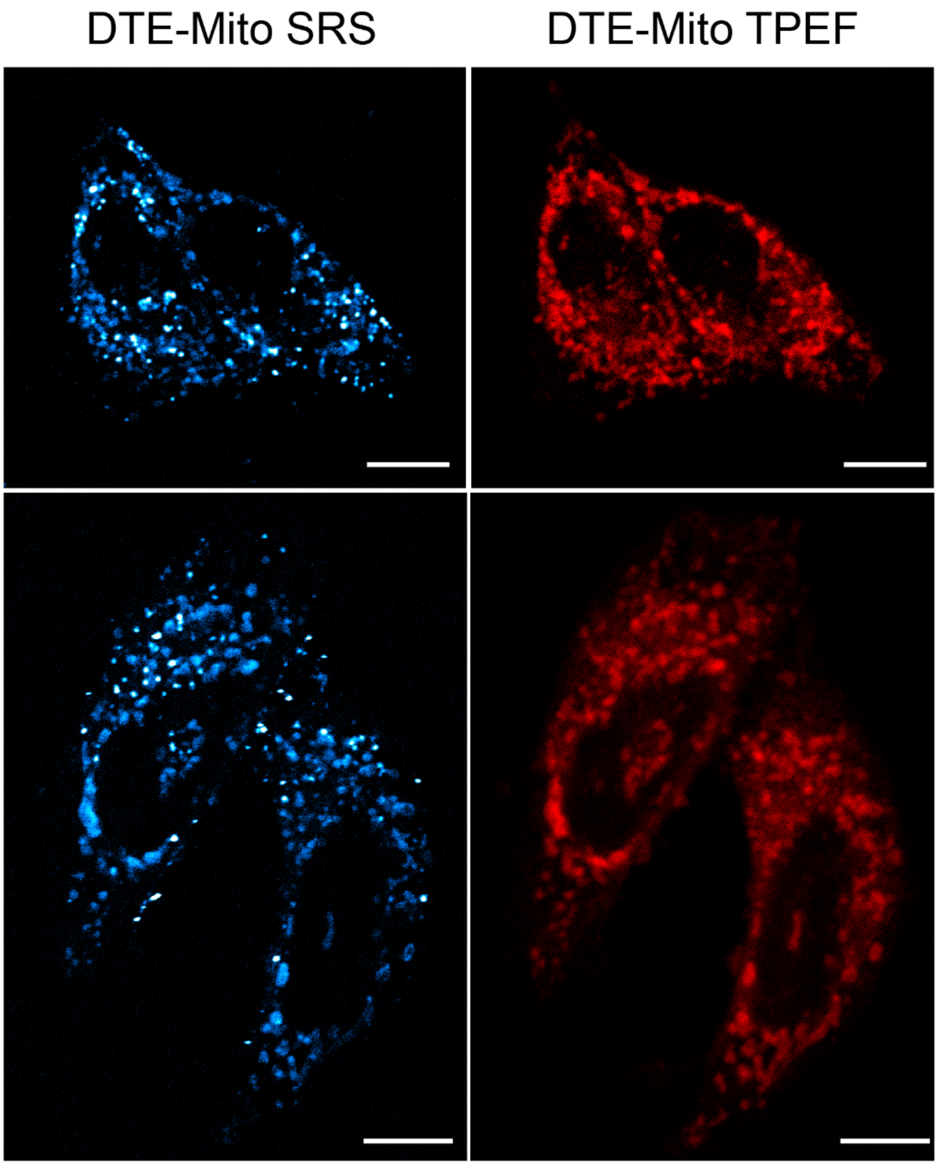

Supplementary Fig. 13. Co-localization images of DTE-Ph-Mito with SRS and TPEF. Scale bar: 10 μm.

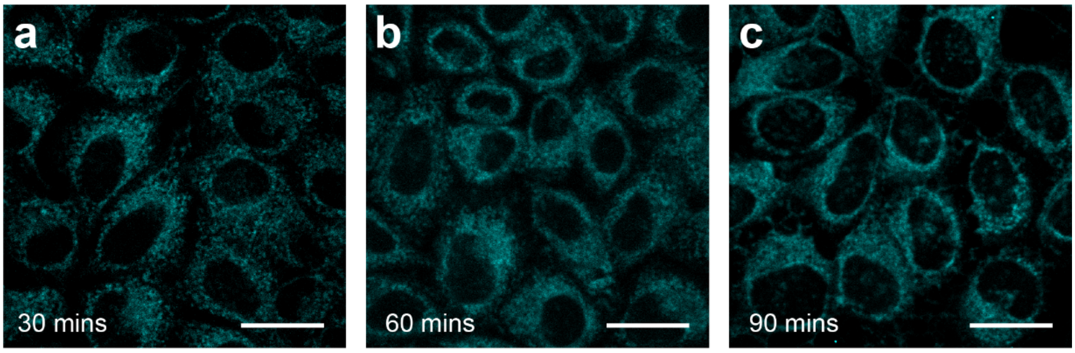

Supplementary Fig. 14. Fluorescence images of HeLa cells stained with 10 μM DTE-Ph-Mito for (A) 30 mins, (B) 60 mins, and (C) 90 mins. Scale bar: 20 μm.

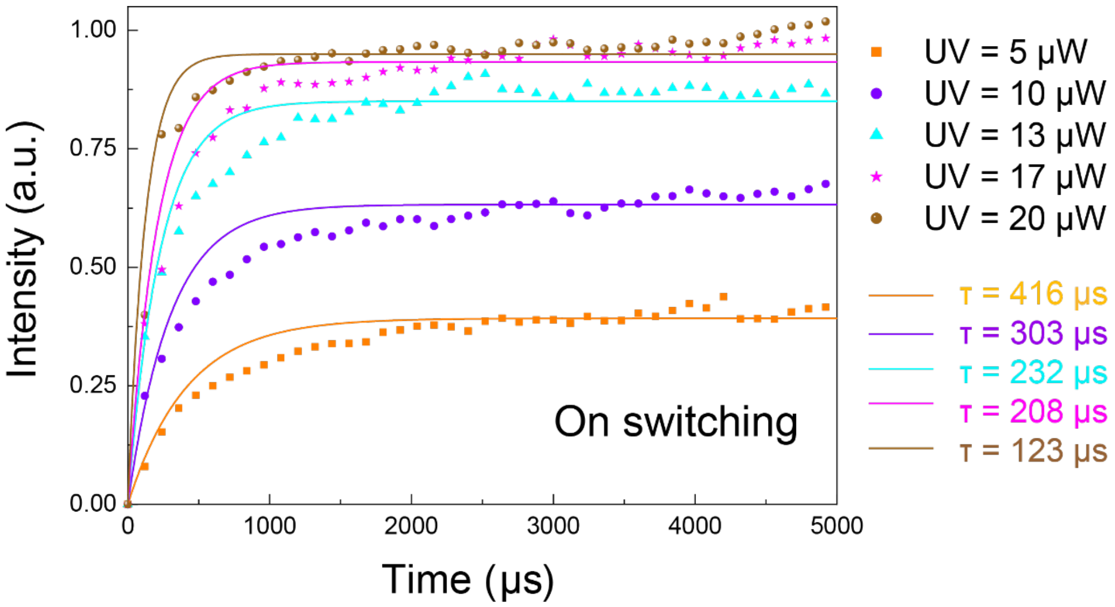

Supplementary Fig. 15. Transition dynamics of SRS intensity triggered by UV pulses.

Supplementary Table 1. Decomposition analysis of the four-layered SRS spectra in Figure 2a.

	closed	open	efficiency	amplification ratio
Layer 4	1.8036	0.8033	0.2084	8.6441
Layer 3	1.0738	0.8634	0.1366	7.8586
Layer 2	0.4235	0.9464	0.0536	7.9026
Layer 1	0	1	0	NA

Reference

1 Fraysse, S., Coudret, C. & Launay, J.-P. Synthesis and Properties of Dinuclear Complexes with a Photochromic Bridge: An Intervalence Electron Transfer Switching "On" and "Off". *Eur. J. Inorg. Chem.* **2000**, 1581-1590, doi:10.1002/1099-0682(200007)2000:7<1581::Aid-ejic1581>3.0.Co;2-2 (2000).

2 Osuka, A., Fujikane, D., Shinmori, H., Kobatake, S. & Irie, M. Synthesis and photoisomerization of dithienylethene-bridged diporphyrins. *J. Org. Chem.* **66**, 3913-3923, doi:10.1021/jo010001p (2001).

3 Ma, J. *et al.* Photoswitching of the triplet excited state of diiodobodipy-dithienylethene triads and application in photo-controllable triplet-triplet annihilation upconversion. *J. Org. Chem.* **79**, 10855-10866, doi:10.1021/jo5018662 (2014).

4 Buchwald, T. *et al.* Identifying compositional and structural changes in spongy and subchondral bone from the hip joints of patients with osteoarthritis using Raman spectroscopy. *J. Biomed. Opt.* **17**, 017007, doi:10.1117/1.JBO.17.1.017007 (2012).
