# Supplementary material for: Switchable stimulated Raman scattering microscopy with photochromic vibrational probes
Source: Nat Commun. 2021 May 25;12:3089. doi: 10.1038/s41467-021-23407-2 (PMC8149663; doi:10.1038/s41467-021-23407-2)
Supplement: Supplementary file 3 — Description of Additional Supplementary Files [file 41467_2021_23407_MOESM3_ESM.pdf]

Description of Additional Supplementary Files

Supplementary Movie 1. Real-time photoswitchable SRS imaging of DTE-Ph powder.
